# The interaction of gut microbiota, genetic variation, and diet in autism spectrum disorder

**DOI:** 10.1002/mlf2.12024

**Published:** 2022-06-26

**Authors:** Xingyin Liu

**Affiliations:** ^1^ Department of Pathogen Biology‐Microbiology Division, State Key Laboratory of Reproductive Medicine, Key Laboratory of Pathogen of Jiangsu Province, Key Laboratory of Human Functional Genomics of Jiangsu Province Nanjing Medical University Nanjing China

The human gut is home to nearly 100 trillion microbes^1^, which constitute the human gut microecological system. The microbiota–gut–brain (MGB) axis is a two‐way communication pathway between gut microbiota and the brain through the immune system, metabolism, and the enteric nervous system. An increasing amount of clinical evidence indicates that abnormal MBG axis communication caused by intestinal ecosystem imbalance can affect the development of many neurological diseases, such as Parkinson's disease, Alzheimer's disease, and autism spectrum disorder (ASD)^2^.

ASD is a neurodevelopmental disorder characterized by social communication deficits and repetitive/stereotypic behaviors according to the Diagnostic and Statistical Manual of Mental Disorders, 5th edition^3^. Ample clinical evidence has shown that children with ASD also display a broad range of nonneurological comorbidities, such as immune, sleep, and gastrointestinal (GI) dysfunction. There are currently no obvious effective medicines for the treatment of ASD. ASD has become a global public health issue. Existing studies have suggested that the interaction between genetic and environmental factors is the main cause of ASD, but its specific etiology remains largely unknown.

## ALTERATION OF GUT MICROBIOTA IN CHILDREN WITH ASD IS ASSOCIATED WITH ABNORMAL BEHAVIOR

In recent years, a series of studies has reported that many children with ASD showed a significant difference in gut microbiota in terms of bacterial diversity, bacterial function, and abundance of specific bacteria compared with healthy children. For example, in 2017, Strati et al.^4^ found a significant increase in the Firmicutes/Bacteroidetes ratio in ASD subjects due to a reduction of the relative abundance of Bacteroidetes. They observed a decrease in the relative abundance of genera, such as *Alistipes*, *Bilophila*, *Dialister*, *Parabacteroides*, and *Veillonella* in children with ASD, while *Collinsella*, *Corynebacterium*, *Dorea*, and *Lactobacillus* were increased. In 2021, through deep metagenomic sequencing of feces samples of 72 children with ASD and 74 healthy children of different ages, Wan et al.^5^ found that the developing dynamics of growth‐associated gut bacteria seen in healthy children was lost in children with ASD across the early‐life age spectrum. Similar to their findings, my group, in collaboration with Yichao Wang and other groups also observed that children with ASD showed a marked but progressive deviation in the development of gut microbiota based on a large cohort aged from 16 months to 19 years^6^. Compared with healthy children, we further found the gut microbiota of children with ASD were distinguished by early unsustainable immature microbiota, altered abundance of bacteria, decreased taxon detection rates, and deregulated microbial metabolic functions with age‐dependent patterns. The altered relationships between microbes were associated with the severity of abnormal behavior, sleep, and GI symptoms in the ASD group. Moreover, based on a quasi‐paired cohort strategy, Zhang et al.^7^ identified the impaired detoxifying function of microbes associated with the gut of children with ASD, suggesting that impaired intestinal microbial detoxification has an effect on toxin accumulation and mitochondrial dysfunction, which is one of the primary pathological manifestations of ASD. Aside from these independent cohort studies on gut microbiota, the effects of microbiota on neurodevelopment and behavior have been demonstrated in various animal models of autism. For instance, Sharon et al.^8^ reported that the transplantation of gut microbiota derived from patients with ASD into germ‐free mice led to autism‐like behavioral phenotypes in these mice and their offspring. The authors highlighted specific bacteria and their metabolites, which can significantly improve behavioral abnormalities and modulate neural excitability of the brain in mouse models of ASD. Collectively, this study demonstrated that gut microbiota can modulate mouse behavior through neuroactive metabolites.

## THE INTERACTION OF HOST GENETIC FACTORS AND MICROBIOME CONTRIBUTES TO AUTISM‐RELATED BEHAVIORAL ABNORMALITIES

There is a consensus that genetic variants, including rare inherited and *de novo* variants, were traditionally believed to be major contributors to individual risk for ASD^9^. As discussed above, the gut microbiome can also influence specific behaviors. This raises the important question of whether the interaction of host genetic variations and microbiome contributes to autism‐related behavioral abnormalities. In 2019, using *Drosophila* as a model^10^, my group showed that one of autism‐risk genes, *KDM5*, caused gut dysbiosis, activation of the immune deficiency (IMD) pathway, and defects of social behavior. We found that administration of antibiotics or feeding with a probiotic *Lactobacillus plantarum* strain partially remedied gut dysbiosis and behavioral defects. Furthermore, *KDM5* was found to transcriptionally regulate a group of genes linking to the IMD signaling pathway and subsequently maintain host‐commensal bacteria homeostasis in a demethylase‐dependent manner. In a recent publication in *Cell* ^11^, Buffington et al. reported that gut bacteria from wild‐type mice could rescue the social deficit of *Cntnap2* (another autism‐risk gene) knockout mice, but could not rescue the hyperactive deficit of *Cntnap2* ^*−/−*^ mice. The authors indicated that the hyperactivity phenotype of *Cntnap2* ^*−/−*^ mice was caused by host genetics, whereas the social‐behavior phenotype was regulated by the gut microbiome. Furthermore, the authors found that the probiotic *Lactobacillus reuteri*, which was deficient in *Cntnap2* ^*−/−*^ mice could improve social deficits in *Cntnap2* ^*−/−*^ mice by elevating the level of tetrahydrobiopterin. The findings indicated that behavioral abnormalities could have different derivations in host genetic variants versus differential bacteria^11^. CHD8 is an autism‐associated protein and may represent a specific genetic subtype of autism. Recently, Fangqing Zhao's group revealed that CHD8 regulated the composition of gut microbiota by modulating α‐defense levels of the intestine^12^, which was associated with increased levels of amino acid transporters in the intestines of *CHD8* ^*+/−*^ mice contributing to the high level of serum glutamine and the increased excitation/inhibition (E/I) ratio in the brain. Impressively, they further found that *Bacteroides uniformis*, which was reduced in *CHD8* ^*+/−*^ mice, improved the ASD‐like behaviors and restored the E/I ratio by reducing intestinal amino acid transport and the serum glutamine levels. Taken together, the above evidence supports the interaction of host genetic variation and gut microbiome contributing to autism‐related behavioral abnormalities.

## THE INTERACTION BETWEEN DIET AND GUT MICROBIOTA MEDIATES NEURODEVELOPMENTAL PLASTICITY

Regarding whether the interaction between diet and gut microbiota mediates neurodevelopmental plasticity, numerous studies have illuminated how abnormal interaction results in different neurodevelopmental outcomes. For example, Buffington et al.^13^ reported that a maternal high‐fat diet (MHFD) induced a shift in microbiota composition that negatively affected offspring social behavior. Social defects and gut dysbiosis in MHFD offspring could be alleviated by cohousing with offspring of mothers on a regular diet through the exchange of microbes. Furthermore, the authors provided strong evidence to support a causal link between maternal diet, gut microbial imbalance, ventral tegmental area plasticity, and behavior. The authors suggested that probiotic treatment may relieve specific behavioral abnormalities associated with neurodevelopmental disorders. Recently, a very insightful study by Mazmanian's group^14^ indicated that dietary tyrosine can be metabolized by the gut microbiota into 4‐ethylphenol (4EP). Furthermore, the authors bioengineered gut bacteria to selectively produce 4‐ethylphenol sulfate in mice. As a result, the mice colonized with 4EP‐producing bacteria showed the activation and connectivity of specific brain regions, disrupted oligodendrocyte maturation and myelination patterns in the brain, and an altered variety of emotional behaviors. In short, the study identified the interaction between the gut and diet‐derived metabolites that contribute to anxiety‐like behaviors.

Taken together, the above studies highlight the role of the gut microbiota–brain axis in the pathology of ASD, which suggests that modifying the gut microbiome may provide therapeutic benefits for patients with ASD. In agreement with the results of basic research, an increasing number of clinical trials targeting the microbiome of patients with ASD via fecal microbiota transplant, probiotics, and prebiotics appear to have potentially positive effects on the behavioral symptoms of ASD^15^, ^16^, ^17^, ^18^, ^19^.

## THE DIFFERENCES IN GUT MICROBIOTA IN ASD PATIENTS MAY REFLECT DIETARY PREFERENCES RELATED TO DIAGNOSTIC CHARACTERISTICS

In contrast to the above research, in a recent publication in *Cell*, Yap et al.^20^ reported that differences in gut microbiota in ASD patients may reflect dietary preferences related to diagnostic characteristics, which raises questions about the existing association between gut microbiota and ASD. This study was based on the cohorts (aged 2–17 years) that included 99 children with ASD, 51 nonsick compatriot (SIB) children, and 97 cases with no blood relationships (UNR). Through fecal metagenome and other clinical data, the authors found that the association between the diagnosis of ASD and the gut microbiota at the species and gene level was weaker than that of age, fecal viscosity, and dietary characteristics. A comparison of ASD with SIB and UNR combinations (covariables: age, sex, and diet) revealed that only *Romboutsia timonensis* showed a significant abundance reduction in children with ASD. Furthermore, the authors found that the dietary diversity and quality were significantly lower in the ASD group than those in the SIB and UNR groups, in sharp contrast to the weak association between ASD diagnosis and diversity of gut microbiota. A negative correlation between dietary diversity and ASD characteristics was also found by Yap et al.^20^ The authors further pointed out that repetitive restrictive behaviors and interests as well as altered sensory perception characteristics of ASD could lead to dietary preference, which is upstream of microbial changes. Taken together, the research suggested that ASD‐related behaviors reduced dietary diversity, mediating the relationship between ASD and gut microbiota. Notably, all psychometric measures were more significantly associated with dietary diversity than microbiome diversity.

Overall, Yap et al.^20^ highlighted the relationship between the manifestation of ASD and dietary preferences, as well as the relationship between dietary preferences and microbiota. This draws exciting attention to the effects of autism‐like behavior on the diet and nutritional status of children with autism. However, the authors ignored evidently accumulated knowledge of MGB^21^, which mediates the relationship between gut microbiota, diet, and behavior as discussed above. For example, studies of the role of gut microbiota in the pathogenesis of ASD should focus on the early life stage linking neural development^22^. Instead, the stool samples in the study were collected from children with ASD aged 2–17 years (average age, 8.7 years), so the data collection time was already missing the critical period of neural development of those under 3 years of age. Moreover, these children generally showed milder ASD symptoms and were not representative of typical children with ASD. Hence, the driving role of microbiota in ASD pathogenesis cannot be ruled out in these cohorts. Furthermore, the authors filtered out a large number of rare bacteria, reducing the amount of useful information on the gut microbiota. As such, the insufficient information weakened the credibility of the conclusion. It must be noted that *R. timonensis*, the identified differential bacteria between children with ASD and healthy children, is significantly associated with repetitive stereotyping. As demonstrated by Mazmanian's group (see above discussion)^14^, a specific microbe‐derived metabolite not microbiota diversity might play an important role in neurodevelopment through its interaction with diet. Hence, future studies should focus on investigating whether the differential *R. timonensis* affects neurodevelopment, which could clarify whether diet‐driven changes in microbiota impact the manifestation of ASD‐related symptoms.

## PERSPECTIVE

**Figure 1:**
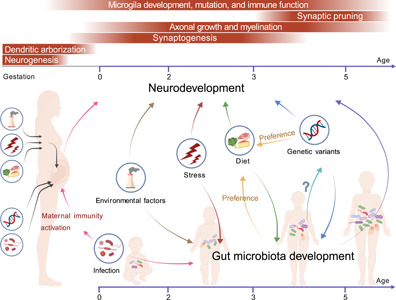
Genetic variants, diet, gut microbiota, and neurodevelopment. Stress, infection, dietary imbalance, exposure to environmental pollutants, and genetic variations from maternal and early life may affect the offspring's development of gut microbiota. An imbalance in gut microbiota might impact different stages of neurodevelopment through the gut–brain axis, mediated by microbial metabolites, the immune system, and the enteric neural system. The imbalance of gut microbiota may induce genetic variations and affect dietary preferences, while genetic variations and dietary preferences could modify the composition of gut microbiota. Moreover, genetic variants and gut microbiota might impact the host's dietary habits, respectively. In addition, genetic variations could directly cause autism‐related behavioral abnormalities by modulating the function of relevant molecular pathways and neural circuits in the brain.

As shown in Figure 1, unlike the host genome, the gut microbiome shows great plasticity and can easily adapt to a variety of environmental and host genetic variation‐derived molecular and metabolite signaling pathway. Among these environmental factors, diet is a key determinant of the composition of gut microbiota. Pregnancy and early life are distinguished by extraordinary alteration in gut microbial composition^23^. Intriguingly, these changes coincide with neurodevelopmental plasticity, including neurogenesis, dendritic arborization, synaptogenesis, axonal growth and myelination, synaptic pruning, microglial development, maturity, and immune function, suggesting a complex dialog between gut microbes and the brain^24^. Epidemiological studies have reported that genetic variation, environmental exposure, stress, lifestyle, unhealthy dietary habits, and infection events from material and early life are all associated with the risk of ASD^20^, ^25^, ^26^, ^27^, ^28^. ASD is a heterogeneous neurodevelopmental syndrome with a complex genetic etiology. However, most of the published studies on gut microbiota in ASD lack patients' genetic information and are relatively small cohort sizes, which may mask the difference in gut microbiota across diverse types of ASD. Future studies based on a longitudinal birth cohort associated with genetic variations are requisite, which could help researchers perform further etiological subtype analyses of ASD. Recently, Satterstrom et al.^29^ identified 102 autism‐risk genes, most of which have roles in regulating gene expression or neuronal communication in early development. Hence, genetic variations could directly cause autism‐related behavioral abnormalities by modulating the function of relevant molecular pathways and neural circuits in the brain. Taken together, exploring the underlying mechanism of how the risk factors interact with each other to contribute to the development of ASD could result in more precise intervention strategies for the treatment of ASD symptoms.
